# A Survey of IoT Security Based on a Layered Architecture of Sensing and Data Analysis

**DOI:** 10.3390/s20133625

**Published:** 2020-06-28

**Authors:** Hichem Mrabet, Sana Belguith, Adeeb Alhomoud, Abderrazak Jemai

**Affiliations:** 1 Department of IT, College of Computing and Informatics, Saudi Electronic University, Medina 42376, Saudi Arabia; h.mrabet@seu.edu.sa; 2 SERCOM-Lab., Tunisia Polytechnic School, Carthage University, Tunis 1054, Tunisia; 3 School of Science, Engineering and Environment, University of Salford, Manchester M5 4WT, UK; 4 Department of Science, College of Science and Theoretical Studies, Saudi Electronic University, Riyadh 11673, Saudi Arabia; a.alhomoud@seu.edu.sa; 5 INSAT, SERCOM-Lab., Tunisia Polytechnic School, Carthage University, 1080 Tunis, Tunisia; Abderrazak.Jemai@insat.rnu.tn

**Keywords:** wearable and non-wearable devices, IoT, communication protocol, security attacks and countermeasures, data analysis

## Abstract

The Internet of Things (IoT) is leading today’s digital transformation. Relying on a combination of technologies, protocols, and devices such as wireless sensors and newly developed wearable and implanted sensors, IoT is changing every aspect of daily life, especially recent applications in digital healthcare. IoT incorporates various kinds of hardware, communication protocols, and services. This IoT diversity can be viewed as a double-edged sword that provides comfort to users but can lead also to a large number of security threats and attacks. In this survey paper, a new compacted and optimized architecture for IoT is proposed based on five layers. Likewise, we propose a new classification of security threats and attacks based on new IoT architecture. The IoT architecture involves a physical perception layer, a network and protocol layer, a transport layer, an application layer, and a data and cloud services layer. First, the physical sensing layer incorporates the basic hardware used by IoT. Second, we highlight the various network and protocol technologies employed by IoT, and review the security threats and solutions. Transport protocols are exhibited and the security threats against them are discussed while providing common solutions. Then, the application layer involves application protocols and lightweight encryption algorithms for IoT. Finally, in the data and cloud services layer, the main important security features of IoT cloud platforms are addressed, involving confidentiality, integrity, authorization, authentication, and encryption protocols. The paper is concluded by presenting the open research issues and future directions towards securing IoT, including the lack of standardized lightweight encryption algorithms, the use of machine-learning algorithms to enhance security and the related challenges, the use of Blockchain to address security challenges in IoT, and the implications of IoT deployment in 5G and beyond.

## 1. Introduction

The Internet of Things (IoT) is considered to be a worldwide network of uniquely addressable interconnected objects, using sensing features, employing communication protocols, exploiting computational capability, and providing services and capacity to analyze data. IoT objects can be doorbells, sensors, Digital Video Recorders (DVRs), light bulbs, electric switches, and home assistant devices. Juniper Research estimates there will be over 46 billion IoT-connected objects by 2021, including devices, sensors and actuators, which represents an increase of 200% compared to 2016 (https://www.i-scoop.eu/internet-of-things-guide/connected-devices-2021/). Near-Field Communications (NFC) and Wireless Sensor and Actuator Networks (WSAN) associated with Radio-Frequency IDentification (RFID) make up the core of the IoT network [1]. The convergence of the Internet and sensor networks is fruitful, and is leading to a new paradigm called machine-to-machine (M2M) communication over the Internet by enabling a very large number of autonomous and self-organized devices [2]. The core concept of IoT is that every object in the network has many capabilities, such as identifying, sensing, and processing data, therefore enabling communication with a wide variety of other devices and services through the Internet to provide services to humanity.

IoT application domains fall into several categories, including utilities, transport and supply chain, environment and agriculture, health, personal home, and manufacturing and industry [3]. Industry 4.0 is a new trend, introducing new technologies to the manufacturing field, such as IoT, cyber-physical systems, big data, cloud computing, the semantic web, and virtualization [4]. As with any trend, many cyber-physical attacks target manufacturers that use Industry 4.0 systems [5], such as the Maroochy water services attack in Australia [6], the steel mill attack in Germany [7], the New York Dam attack [8], and the Norwegian Hydro aluminum attack (www.bbc.com/news/technology-47624207) in 2000, 2014, 2016, and 2019, respectively.

IoT, being an emerging technology as well as having huge number of devices deployed and connected to the Internet, represents a fertile field for attacker threats, and therefore new cyber-security issues related to IoT have appeared. Many threats threatening IoT devices have been 2 defined, including network, physical, environment, cryptanalysis, and software attacks [9]. Network attacks include man-in-the-middle (MITM), replay, masquerade, and distributed denial of service (DDoS) attacks [10]. To overcome these risks to IoT systems, communication protocols should be secure, lightweight encryption algorithm should be implemented, IoT platform security features should be enforced, and advanced techniques should be applied to filter and predict different security threats.

Security in IoT is of extreme importance, as any successful attack may paralyze a whole manufacturing, transport, health system, etc. sector. IoT is a combination of devices, network protocols, and technologies that each have their own vulnerabilities, which increases the attack surface across the whole IoT network. In other words, several attacks against IoT have been inherited from underlying technologies.

**Contributions**—There has been no standard until now for IoT architecture. However, different architectures have been proposed for IoT, such as three-layer [11], middle-ware-based architecture [12], service-oriented architecture (SOA) [13,14], four-layer [15], and five-layer [12].

Architecture previously proposed in the literature is highlighted in this paragraph. The basic model is called three-layer architecture, and it is composed of perception, network, and application layers [11,12,16]. Four-layer architecture covers perception, network, middleware, and application layers [13,15,16]. The role of the middleware layer involves service management, data storage, and service composition [15]. A proposed five-layer architecture includes objects, object abstraction, service management, application, and business layers [12].

**Figure 1:**
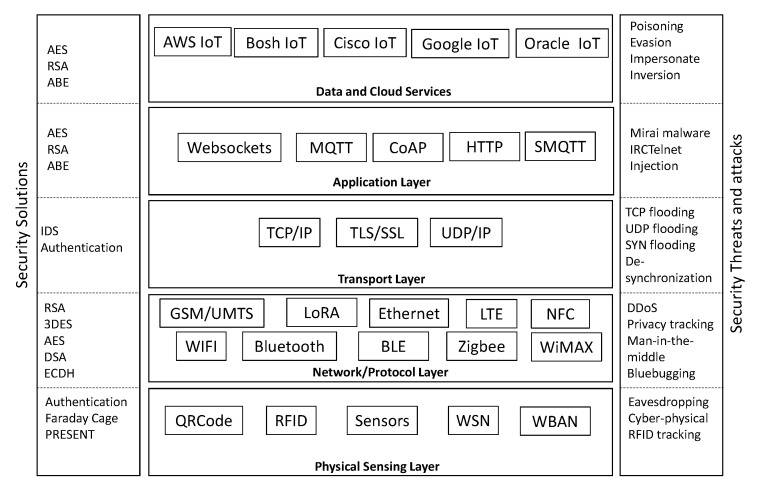
The proposed IoT architecture.

To add advanced features to IoT such as IoT data, machine-learning algorithms, and light encryption algorithms, we propose in this paper a new IoT architecture, as shown in Figure 1. The proposed IoT architecture is based on five layers, including a perception layer, a network/protocol layer, a transport layer, and a data and cloud services layer. As shown in Figure 1, the physical layer involves different sensors and IoT devices such as a Wireless Sensors Network (WSN), QR Codes, Wireless Body Area Network (WBAN), Radio-Frequency IDentification (RFID) devices, etc.

The network and protocol layer covers different wired and wireless network protocols involved in an IoT system, such as Wi-Fi, ZigBee, Ethernet, Bluetooth, LTE, 5G, etc. The transport layer involves TCP/IP, UDP/IP, and Transport Layer Security (TLS)/secure sockets layer (SSL) suite protocols. For the application layer, we cover the various application protocols developed to meet the IoT requirement in terms of low power consumption and small device capacity, such as Advanced Message Queuing Protocol (AMQP), Constrained Application Protocol (CoAP), and Message Queuing Telemetry Transport (MQTT). Finally, the data and cloud services layer presents the main cloud-based IoT frameworks.

In Table 1, common IoT attacks are highlighted. We also provide security control suggestions to mitigate the harm to IoT devices caused by these attacks.

The paper focuses on analyzing security issues inherited by each layer component, while presenting deployed security measures and mechanisms to defeat prominent attacks.

As shown in Table 1, common IoT attacks can be classified into 5 classes:

- Data and cloud services layer attacks include poisoning, evasion, impersonation, and inversion.
- Application layer attacks include Mirai malware, IPCTelnet malware, DDoS, and injection.
- Transport layer attacks include resource exhaustion, flooding, replay, DDoS attack, and amplification attacks.
- Network and protocol layer attacks include man-in-the-middle, DDoS, and replay attacks.
- Physical sensing layer attacks include eavesdropping, cyber-physical, and tracking attacks.

A scenario to describe the realistic use of the proposed architecture could be an e-health application, in which the perception layer captures a physical parameter via a sensor implemented in a patient’s body. Then, the job of the network and transport layers is to send the data to the application layer by selecting the suitable communication and lightweight encryption protocol based on power processing and energy consumption of the IoT device. The application layer will select the appropriate application protocol (i.e., MQTT, CoAP, or other) to communicate the data to the right user (i.e., doctor or medical staff). Finally, the data will be stored in the cloud layer and will be useful for future data analysis and prediction by using the appropriate machine-learning algorithm.

**Existent Surveys**—Internet of things security issues have attracted a lot of research, in which several published survey papers have studied IoT architecture, applications, and security issues. The survey authored by Al-Fuqaha et al. [12] covers the main IoT element-enabling technologies and the principle common IoT standards. In [11], the authors address the security of IoT frameworks such as AWS, Azure, and Calvin architecture. The authors in [16] provide a survey of the most common architectures proposed for IoT e-health applications, smart society applications, and cloud service and management solutions. Moreover, [4] addresses IoT in terms of the requirements of smart factories to enable standard Industry 4.0 protocols in the next industrial revolution. Key IoT applications in industries are presented in [13] including the food supply chain, the iDrive system provided by the BMW car company, and an environment monitoring system for firefighting based on RFID tags. Buton et al. [17] introduced a security analysis of IoT based on an in-depth analysis of the use of WSNs, their vulnerabilities and their major security threats. Recently, Hussain et al. [18] presented a review of machine learning applied in IoT, and their main advantages and limitations.

**Position of our paper**—In this survey paper, we combine different aspects related to IoT technologies in one compact IoT architecture, covering IoT physical devices and sensors, communication and network protocols, a transport layer, an application layer, and data and cloud services. This architecture is based on a modification of OSI architecture, considering the security vulnerabilities and threats. In addition to existent OSI layers, we define a cloud and data layer, which involves several publicly available IoT frameworks providing IoT data storage, processing, and analysis. This architecture is extended to involve machine-learning applications that process data and protect IoT components. Furthermore, we present a discussion of current challenges facing IoT security solutions, such as the lack of standard encryption algorithms adapted for IoT devices. We also explore the application of novel techniques to secure IoT, such as the use of Blockchain in IoT and machine-learning models, as well as reviewing the potential of 5G network applications, and their reliance on IoT.

**Paper Organization**—This survey is organized as follows. Section 2 presents the main components of the physical sensing layer, and the related security threats and countermeasures. The IoT network and communication protocols and their related security issues and solutions are reviewed in Section 3. Section 4 introduces an overview of the transport layer protocol and its main security countermeasures. The application layer protocols are studied in Section 5, detailing their main security features. Section 6 reviews the well-known cloud-based IoT frameworks, while reviewing the main security measures they are implementing. Finally, a discussion of open issues and research opportunities is conducted in Section 7, before the survey paper is concluded in Section 8.

## 2. Physical Sensing Layer

### 2.1. Underlaying Technologies

The components of the physical sensing layer mainly involve but are not limited to QR codes, sensors, RFIDs, WSANs, and WBANs. In the state of the art, RFID uses a universal unique identifier called an Electronic Product Code (EPC) to identify objects in the IoT network. It supports various applications in several areas, such as logistics and supply-chain management, aviation, food safety, retailing, and public utilities. Likewise, the RFID system is characterized by its small size, very low cost, and no limitation to battery life. The second element that defines the core of the IoT network is WSAN, which can provide high radio coverage and communication paradigm, is peer-to-peer, while wireless sensor networks support sensing, computing, and communication capabilities in a passive system [1]. However, IoT benefits from the tracking capabilities offered by RFID tags [2]. WBAN stands for the wireless body area network and is defined as a set of sensors implemented in a patient’s body to capture health parameters, including temperature, blood pressure, and glucose rate. The different sensors communicate the human vital signals to a health monitoring system via Bluetooth or ZigBee protocol.

### 2.2. Security Threats and Solutions

RFID is described by ISO/IEC 18000. However, RFID suffers from weak privacy. In addition, physical threats to RFID system disable tags, modify their content, and imitate them [19]. According to [9], a Faraday cage, tag-killing, tag-blocking, and re-encryption are effective solutions for RFID tracking attacks.

In the state of the art, the three kinds of attacks against the perception layer are eavesdropping, cyber-physical, and RFID tracking. An eavesdropping attack, also called a sniffing or snooping attack, occurs when someone tries to pick up information sent by IoT devices via a network. A cyber-physical attack happens when a sensor in a WSN is physically attacked or compromised by a cyber-attack (called a faulty node). Various solutions have been proposed to overcome this attack, such as using a localized fault-detection algorithm to identify the faulty nodes in WSN [20], using a decentralized intrusion-detection system model for the WSN [21], and introducing a derived intrusion-detection probability in both homogeneous and heterogeneous WSNs [22]. A RFID tracking attack attempts to disable tags, modify their contents, or imitate them. Various security solutions are proposed to overcome this attack, such as using a localized fault-detection algorithm to identify the faulty nodes in the WSN [23], using a decentralized intrusion-detection system model for the WSN [21], and introducing a derived intrusion-detection probability in both homogeneous and heterogeneous WSNs [24]. Physical threats to the RFID system are disabling tags, modifying their content, and imitating them [19]. According to [9], a Faraday cage, tag-killing, tag-blocking, and re-encryption are effective solutions against eavesdropping and RFID tracking attacks. RFID is described by ISO/IEC 18000. In addition, a Faraday cage is one of the effective solutions for RFID consumer privacy against eavesdropping and tracking attacks [9]. Since WBAN uses wired and wireless protocol to communicate sensitive patient data, it can be vulnerable to malicious attacks such as eavesdropping, spoofing, and tampering, leading to a compromise of the privacy of the protected health information system [25]. Various solutions have been proposed in the literature to enforce access control and security communication between WBAN and external users (i.e., doctors and medical staff) such as the cyphertext policy attribute-based encryption (CP-ABE) where access is granted to the user who has at least d out of n attributes of the patient-related data [26,27].

## 3. Network and Protocol Layer

### 3.1. Underlaying Technologies/Background

Communication protocols are a main component of the IoT systems, enabling the establishment of communication and exchange of data between IoT devices and other distant parts of the network. The network and protocol layer includes ZigBee [28], 3G/4G/5G wireless communication [29], Wi-Fi [30], and Bluetooth [16]. In Table 2, we address the standard security feature (i.e., encryption protocol and key length), and advantages and disadvantages for the most relevant data-link communication protocols. Some research works divide IoT communication protocols into two sub-layers—sensor-based network and gateway network [16].

The sensor-based network relies on different protocols used by devices to communicate between each other. These protocols include but are not limited to Bluetooth, Bluetooth Low Energy (BLE), Worldwide Interoperability for Microwave Access (WiMAX), Wi-Fi, ZigBee, etc. [28,30]. The gateway network is responsible for routing data from/to a low-power lossy network (LLN) to/from the Internet or a close-by Local Area Network (LAN). These protocols include Ethernet, 3G/4G/5G, 6LoWPAN, etc. [29,31].

Various basic communication protocols are used in IoT networks to ensure communication among all objects for wired and wireless networks. Bluetooth is described by the IEEE 802.15.1 standard. In its 4.2 version, Bluetooth uses the Federal Information Processing Standard (FIPS)-compliant elliptic curve Diffie–Hellman (ECDH) algorithm for key generation (i.e., Diffie–Hellman key, or DH key). However, Bluetooth suffers from easy privacy/identity tracking. Wi-Fi is described by the IEEE 802.11i/e/g standard and it can support AES 128 key length. Mobility and efficiency are the most important benefit, while limited reachability (i.e., in the range of 100 m) is the main disadvantage [12]. ZigBee presents low-cost, low-energy devices, and one-time transmission of the unprotected key as an advantage and a disadvantage, respectively [9]. WiMAX is described by the IEEE 802.16 standard, which is a collection of wireless broadband standards. WiMAX provides data rates from 1.5 Mb/s to 1 Gb/s. NFC technology was developed by Philips and Sony in 2002 to provide contactless communication [32]. NFC is a short-range half-duplex communication protocol. NFC relies on coupling between the receiver and the sender. NFC works within a few centimeters under an operating frequency equal to 13.56 MHz. 3G and 4G mobile communication protocols are standardized by the universal mobile telecommunications system (UMTS) and Long-Term Evolution (LTE), respectively. IPv6 over LoWPAN (6LoWPAN) is a low-cost communication network allowing wireless connectivity between devices with limited power and processing capability. A 6LoWPAN typically includes devices that work together to connect the physical environment to real-world applications, e.g., wireless sensors. 6LoWPAN is standardized by the IEEE 802.15.4-2003 standard (IEEE802.15.4).

### 3.2. Security Threats and Countermeasures

Several common attacks have been launched against IoT communication protocols in which the attack can target most communication protocols such as eavesdropping against Bluetooth, NFC, Wi-Fi, etc. [33]. Man-in-the-middle attacks and Denial of Service (DoS) attacks also can be launched against various IoT communication protocols. To address different attacks, such as eavesdropping and replay attacks, RSA and Diffie–Hellman algorithms are the emergent solution for LTE-advanced (LTE-A)’s security features [34]. Some other attacks are dedicated to specific protocols, such as attacks against Bluetooth that are defined as follows:

- Bluejacking: This is the use of Bluetooth for sending unsolicited messages to other enabled devices. This attack exploits the Object Exchange (OBEX) protocol which is used by Bluetooth-enabled devices for exchanging data and commands [35].
- Bluebugging: This is an attack where the attacker exploits devices by manipulating the devices into compromising its own security, leading to unauthorized access of the device. The Bluebug attack focuses on or uses AT Commands (ASCII Terminal) when performing attacks [36,37].
- Bluesmack: This is an attack that causes denial of service to Bluetooth devices. This attack sends a Logic Link Control and Adaptation Protocol (L2CAP) ping request, which is similar to the ICMP ping attack, leading to devices being knocked out after receiving an oversized packet, which in turn leads to a DoS [38].

Since smart objects have a limited calculation capacity, restricted energy, and limited memory, lightweight encryption algorithms are widely used in the IoT field, such as in RFID tags, sensors, and healthcare devices [39]. Additionally, the lightweight concept for IoT is extended to lightweight attribute-based encryption schema for cloud applications [40,41,42], lightweight collaborative key management protocol [43], lightweight protocol for smart home authentication and key-session exchange [44,45]. Many IoT protocols have been proposed for different ISO layers, such as link layer (802.15.4, PLC), network layer (RPL, 6LoWPA), presentation layer (TLS, 802.1AR, 802.1X), and application layer (CoAP) [46]. Since 6LoWPA takes advantage of the IEEE 802.15.4 standard for low-rate wireless networks and IPv6, it provides low processing and a lack of authentication as an advantage and a disadvantage, respectively [9]. RPL uses the Advanced Encryption Standard (AES) protocol with key length of 128 [47]. RPL can support point-to-point communication and multi-cast routing in lower power networks [46]. However, its vulnerability to many attacks, such as forwarding, sinkhole, Sybil, Hello flooding, wormhole, black hole, and DoS, is the greatest disadvantage of RPL [9]. NFC is described by the ISO/IEC 14443 standard and it can support various cryptosystems including RSA, digital signature algorithm (DSA), and elliptical curve digital signature algorithm (ECDSA) with a key length of up to 128 [48]. However, it presents a limited range between different active readers. A common attack in the network layer is the man-in-the-middle (MIM) attack. Two effective solutions for preventing MIM attacks are the use of an Intrusion-Detection System (IDS) and a Virtual Private Network (VPN). With the increasing use of IoT, botnet infections targeting IoT devices have become a noticeable threat. IoT devices suffered from a powerful botnet infection in 2016 due to the Mirai botnet malware [49]. According to [50] the latter botnet could infect and take control of more than 49,000 IoT devices distributed across 164 countries. Alhomoud el al. [49] identify botnets as a cluster of nodes infected by the same malware, where each node can serve as a bot (derived from the word robot) and is capable of performing certain actions or executing commands automatically, and mimicking human activates. One of the most common uses of botnets is to launch DDoS attacks. DDoS is an attempt to make a machine or network resource unavailable for its intended use to break the availability of a system or the network. Ingress/Egress filtering, D-WARD, Hop Count Filtering, and SYN-Cookies are DDoS attack countermeasures [23].

## 4. Transport Layer

### 4.1. Underlaying Technologies

The transport layer offers two services—a connection-oriented protocol, named TCP, for reliable application, and connectionless protocol for unreliable applications. TCP uses TLS to ensure a secure transport layer. However, UDP uses DTLS to secure the transport layer. By default, the lightweight connectivity protocol MQTT does not include a security layer. Therefore, the user is responsible for defining a security protocol, either TLS or SSL, and to enable a certificate and session key management [17]. Likewise, TLS and SSL are vulnerable against various kinds of attacks such as BEAST, CRIME, Heartbleed, and RC4. The basic form of MQTT, without a security protocol and with the weakness of TLS and SSL, is called an MQTT exploit.

### 4.2. Security Threats and Solutions

One of the most important weaknesses of the transport layer in IoT is the vulnerability of the TLS protocol to resource exhaustion, flooding, replay, and amplification attacks. A replay attack happens when the intruder manipulates a message stream and maliciously reorders the data packets to change the meaning of the message [28]. To protect IoT devices from a replay attack, setting the timeliness of the message is an effective security control. A DDoS attack can be considered to be a network/transport and application layer attack. The taxonomy of attacks against the transport layer caused by the DDoS is classified into TCP flooding, UDP flooding, TCP SYN flooding, and TCP desynchronization. TCP flooding and UDP flooding consist of sending many packets through the TCP and UDP protocol to stop or to reduce its activities. TCP SYN flooding is can open an external connection without respecting the TCP handshake procedure. TCP desynchronization, also called TCP hijacking, is defined as an attempt to break the packet sequence by injecting it with a wrong sequence number. In the state of the art, two solutions have been proposed to overcome the TLS issue. One is to use DTLS, and the other is to use an end-to-end tunnel to protect a low = power and lossy network [28]. Recently, various proposed solutions based on machine learning (ML) to detect DoS and DDoS have been proposed in the literature, such as the unsupervised clustering model, the Linear Vector Quantization (LVQ) model of Artificial Neural Network (ANN), and the Back-Propagation (BP) model of ANN. A pertinent classifier based on Support Vector Machine (SVM) to detect and prevent DDoS TCP flooding attacks upgrades the K-nearest, naive Bayes, and multilayer perceptron in terms of performance [51]. Finally, one effective solution against the MQTT exploit is to secure the MQTT protocol by implementing the attribute-based encryption through the elliptic curve [52].

## 5. Application Layer

### 5.1. Underlaying Technologies

Our IoT architecture application layer includes application protocols. Various application protocols have been developed to meet the IoT requirement in terms of low power consumption and small device capacity such as Advanced Message Queuing Protocol (AMQP), Constrained Application Protocol (CoAP), Data Distribution Service (DDS), and Message Queuing Telemetry Transport (MQTT). MQTT is a specific application protocol that potentially enhances machine-to-machine communication between a client and a server. MQTT protocols can work under various data-link layer protocols, such as Ethernet and Wi-Fi. Additionally, they are characterized by being very lightweight and are a very effective solution to exchanging small messages between a broker (i.e., a server) and nodes (i.e., clients). Currently, the most important challenge for MQTT is adaptation to emergent technologies, such as LTE, 5G wireless, and mobile communications. Several advantages have been provided by MQTT, such as routing for small, cheap, low-power and low-memory devices in low-bandwidth and vulnerable networks [12]. MQTT was standardized in 2013, and presents three QoS levels. Likewise, an extension of MQTT is called Secure MQTT (SMQTT), and was proposed to tackle security issues. This extension is based on TCP/IP Internet suite protocol as depicted in Table 3. Many applications use MQTT, such as healthcare and Facebook notifications.

### 5.2. Security Threats and Solutions

Mirai malware, IRCTelnet, and injection are the common IoT attacks in the application layer. Mirai malware attack happens when a hacker tries to gain access to an IoT device by using a default Telnet or SSH account [53]. Therefore, to stop these attacks, the default accounts of Telnet and SSH should be disabled or changed. Likewise, IRCTelnet is based on forcing a Telnet port to infect the LINUX operating system of an IoT device [54]. One security measure to prevent an IRCTelnet attack is to disable the Telnet port number. According to the 2017 OWASP application security flaws review, the ten most critical web application security risks are: injection, broken authentication, sensitive data exposure, XML external entities (XXE), broken access control, security misconfiguration, cross-site scripting (XSS), insecure deserialization, and using components with known vulnerabilities (www.owasp.org, OWASP Top 10—2017 The Ten Most Critical Web Application Security Risks). Furthermore, injection is defined as untrusted data that is sent to an interpreter as part of a command or query to bring down the application using this data. An effective security control to prevent the user from entering more or less than the required format, and to prevent a hacker from abusing an application system, is input validation control [55]. SMQTT is proposed to improve MQTT security characteristics based on lightweight encryption. Many papers have proposed various versions of MQTT to enhance security features by adding encryption algorithms such as AES and Rivest–Shamir–Adleman (RSA) [56]. The security of the communication for SMQTT is provided through widespread SSL and transport layer security (TLS) protocols. In the state of the art, many variations of TLS, such as wireless TLS (WTLS) and datagram TLS (DTLS), are used in mobile communications and UDP-based applications, respectively, to ensure data privacy and integrity. In this section, we highlight the security protocols most used in IoT communication to ensure data confidentiality and privacy. Data confidentiality is guaranteed by encryption protocols. Additionally, data is sanitized, and privacy is preserved. Table 4 presents the most important lightweight encryption algorithms for IoT in terms of key size, average execution time of 1000 iterations, and applications for both symmetric and asymmetric algorithms. Symmetric cipher algorithms support message integrity checks, encryption, and entity authentication. Additionally, asymmetric cipher algorithms provide non-repudiation and key management [57].

PRESENT is a symmetric lightweight algorithm using a 64-bit block with 80/128-bit key length [58]. In addition, CLEFIA is proposed in the ISO/IEC 29192-2 light cryptography standard, the CRYPTREC project for the revision of the e-Government-recommended ciphers list in Japan, and it is employed by the Sony Corporation for digital rights management [59]. Additionally, RSA and elliptic curve (EC) are asymmetric lightweight algorithms. Moreover, RSA uses common public-key cryptography algorithms, and EC is very useful in pervasive computing [60,61]. Furthermore, three variants of EC algorithms are implemented—ECDAC for digital signature, ECIES for data encryption, and ECDH for key exchange [46].

## 6. Data and Cloud Services Layer

The development of applications for IoT faces many challenges due to the complexity of distributed computing, the involvement of different programming languages, and the variety of communication protocols. Therefore, the development of IoT applications requires the management of both hardware and software components, along with the handling of full infrastructure and delivery of functional and non-functional requirements. These challenges have led to the emergence of a cloud-based IoT programming framework launched by the major IoT stakeholders to provide ready-to-use/develop IoT applications.

The cloud-based IoT frameworks introduce a set of rules and protocols aimed at organizing data management and message exchange between the parties involved in the IoT network, such as devices, the cloud system, and users. These frameworks enable a simplified high-level deployment of IoT applications while hiding the complexity of the underlaying protocols.

In this section, we review the performance of the five main IoT frameworks based on public clouds, namely Amazon AWS IoT, CISCO IoT Cloud Connect, Google Cloud IoT, Oracle IoT Ecosystem, and Bosch IoT Suite. We have chosen these frameworks in the absence of a standardized framework, as they are the best-known ones. We focus on reviewing the security features provided by these frameworks as well as the inherited security threats by using public cloud architecture.

The cloud-based IoT frameworks are built on three main components: smart devices such as sensors, tags, etc., the cloud servers providing storage and processing of IoT data, and the users represented by the applications that access cloud-stored data and communicate with the devices. The frameworks also include the protocols that are needed to communicate between all the entities.

In Table 5, we compare the security features provided by the selected IoT frameworks. Providing a secure framework relies mainly on ensuring confidentiality, integrity, availability, authentication, and access control [55].

To ensure secure communication while transferring and accessing IoT data, various protocols are used by the aforementioned IoT frameworks, including Hypertext Transfer Protocol Secure (HTTPS), IPsec, transport layer security (TLS), datagram transport layer security (DTLS), and MQTT over TLS. Basically, SSL is used by AWS, Google Cloud and Oracle IoT Ecosystem.

AWS IoT is composed of four components, namely the device gateway, the rules engine, the registry, and the device shadows (https://docs.aws.amazon.com/iot-device-management/index.html). The device gateway is an intermediate component enabling communication between devices and cloud services via the MQTT protocol. The rules engine is responsible for processing the exchanged messages to forward them to the AWS, the subscribed devices, or a non-AWS service. The registry unit assigns an identifier to every connected device, while storing metadata to enable their tracking. The device shadow is a virtual device image created and stored in the cloud, enabling the saving of the last online state of the device and enforcement of future changes to the state once it goes online again. In a nutshell, the framework enables the management of IoT devices using its shadow even when it is not connected to the network.

To ensure confidentiality, integrity and availability, AWS proposes SSL-protected API endpoints (https://docs.aws.amazon.com/iot-device-management/index.html). AWS security modules ensure authentication and authorization. AWS authentication is based on X.509 certificates. On the other hand, AWS authorization is based on identity and access management (users, groups, and roles). Additionally, AWS Cognito identity modules are used to create unique user identities [11].

Google Cloud uses three kinds of encryption protocols to ensure the protection of data at the application layer. These are AES, TLS and secure/multipurpose Internet mail extensions (S/MIME) (http://cloud.google.com/security/encryption-in-transit). Likewise, Google cloud uses application layer transport security (ATLS) to guaranty confidentiality, integrity and authentication among different services. Also, Google Cloud suggests various access control options, such as cloud identity and access management as well as access control lists (ACLs).

The Oracle IoT solution is based on transparent sensitive data protection (TSDP) to ensure confidentiality and integrity. In addition, to improve data security, Oracle employs data masking and sub-setting to comply with the payment card industry data security standard (PCI-DSS) (www.oracle.com/technetwork/database/security/security-compliance).

CISCO IoT platform architecture is composed of four layers. These are an embedded systems and sensors layer, a multi-service edge layer, a core layer, and a data center cloud layer. The core layer includes IP/MPLS, security management, and network service. CISCO proposes an IoT/M2M security framework. Strong authentication is well provided by using AES and RSA for digital signature and key transport (www.cisco.com/secure-iot-proposed-framework, CISCO Kinetic Security Technical Paper). To ensure secure data traffic and data management, The CISCO Cloud solution employs HTTPS over IPsec, and SNMP over IPsec, respectively. Likewise, authorization and access control in CISCO IoT Cloud Connect uses segment data based on destination.

The architecture of the Bosch IoT suite expects an identity management module for users, roles, relations, and permissions. Regarding Bosch cross-domain applications (i.e., case of XDL120), confidentiality and integrity are based on the Wi-Fi-protected access 2 (WPA2) provided by the standard IEEE 802.11i/e/g white-listing of MAC addresses (https://www.digikey.co.uk/en/supplier-centers/b/bosch-cds). Furthermore, XDL120 employs DTLS to ensure a secure communication of transmitted sensor parameters and lightweight M2M (LWM2M) communication protocols.

In addition, cloud-based IoT frameworks provide access to machine-learning functions, enabling the processing of collected IoT data.

Research has identified multiple applications of machine learning in IoT contexts. The taxonomy of ML in IoT contexts for big data analysis is presented in Table 6. These ML models are categorized into three categories—classification, regression, and clustering [62]. The ML classification family includes K-Nearest Neighbors (KNN), Naive Bayes (NB), and SVM. The ML clustering family involves K-means, a density-based approach to spatial clustering of applications with noise (DBSCAN), and the Feed Forward Neural Network (FFNN). The ML regression family covers Linear Regression (LR) and Support Vector Regression (SVR).

One important application of KNN clustering machine learning is to enable smart tourism and tourist pattern tracking. Then main advantage of KNN is that the online settings are easy to update; however, KNN is unscalable to large datasets. NB is applicable in many fields, such as spam filtering, text categorization, and automatic medical diagnosis [63]. Due to applying Bayes’ theorem with the “naive” assumption of independence between the features, Naive Bayes classification is fast and highly scalable. The most important application of SVM is real-time prediction, which makes it suitable for real-time intrusions and attack detection. In addition, SVM has the capability to deal with high-dimensional datasets. Nonetheless, SVM suffers from a lack of transparency of results. LR can process at a high rate [64], and this algorithm is useful in many applications, such as economics, market analysis, and energy usage (to analyze and predict the energy usage of buildings, for example). However, LR is very sensitive to outliers. SVR uses the same basic idea as SVM, a classification algorithm, but applies it to predict real values rather than a class. SVR informs the presence of data non-linearity, and a prediction model is provided. Additionally, SVR is a useful and flexible technique, helping the user to deal with limitations pertaining to the distributional properties of underlying variables (https://rpubs.com/linkonabe/SLSvsSVR). The applications of SVR include the forecasting of financial markets, prediction of electricity prices, estimation of power consumption, and intelligent transportation systems [65]. The K-means clustering algorithm is present in many IoT applications, such as smart city, smart home, smart citizen, and air traffic control [66]. The most important benefits of K-means includes the high scalability and speed. However, K-means presents various disadvantages such as difficulty in predicting the number of clusters (K-Value), and sensitivity to scale. DBSCAN is an effective ML clustering algorithm, especially for large datasets. In addition, DBSCAN is very suitable for smart cities and for anomaly detection in temperature data applications [67]. Nonetheless, in the case of a dataset with large differences in densities, the clustering process is not efficient. Likewise, the performance of the model is sensitive to the distance metric used for determining whatever region is dense [68]. FFNN is a neural network trained with a back-propagation learning algorithm. The major advantages of FFNN are its adaptability without support of the user, non-linearity, and robustness. FFNN suffers from having a high number of weights in the neural network and requiring a longer time for training. The application fields of FFNN are smart health and chemistry (i.e., for the prediction of multi-state secondary structures).

The Generative adversarial network (GAN) is a pertinent type of machine learning that is receiving increased attention from researchers, based on two networks—generative network and discriminative network. The first network is used to generate new candidates from a known dataset, while the second serves as candidate evaluation. New emergent applications of GAN are applied in various fields, such as semi-supervised salient object detection in cloud-fog IoT devices [69] and high-resolution image generation [70]. On the other hand, the Floor of Log algorithm associated with KNN and SVM is a promising supervised technique based on compressed features for power reduction of mobile devices running face-recognition applications [71].

## 7. Open Research Issues and Future Directions

Ensuring a fully secure IoT network is still a challenge that can hold back complete adoption of IoT application in daily life. There are multiple open issues and challenges to the provision of more secure IoT networks that constitute great opportunities for researchers. The first deciding factor in terms of security that will shape the future direction of IoT is the building of a standard architecture to ensure secure and reliable communication from a perception layer until cloud layer-like TCP/IP architecture in an Internet context. The second factor is the specification and selectin of the required lightweight encryption algorithm that fulfils IoT device capacity in terms of processing power and energy consumption. In this section, we review some future directions that will enable secure and private IoT application by either developing dedicated solutions, or adopting novel application of existing technologies.

### 7.1. The Lack of Standardized Lightweight Encryption Algorithms for IoT Applications

Efforts are being made to define a standard for lightweight encryption algorithms that are designed for IoT applications. Many requirements need to be fulfilled as IoT devices are resource-constrained devices. The main obstacles for proposing lightweight security algorithms for all IoT applications are the limited capacity of IoT devices in terms of energy consumption, processing power, and memory capacity. A minimum requirement for each lightweight security algorithm should be defined, such as key size, energy consumption, and execution time. Several encryption algorithms have been designed to suit IoT applications. Conventional algorithms have been applied to secure IoT including tiny encryption algorithm (TEA), which provides lower memory use and ease of implementation on both hardware and software scales [72]. AES has been also adopted to provide secure communication between IoT devices [73]. Though an attribute-based encryption algorithm requires high computation costs, several lightweight versions have been designed to suit IoT applications, such as reduced computation algorithms [40,74], offloading heavy computations to an edge [75], or cloud server [26].

### 7.2. Use of Machine Learning to Enhance Security in IoT

Recently, there has been an increased interest in targeting the use of machine-learning models to secure IoT applications [76].

Meidan et al. proposed [77] a Random Forest model, which is a supervised machine-learning algorithm, to extract features from network traffic data to detect unauthorized IoT devices.

Distributed Denial of Service (DDoS) attacks are increasing against IoT networks with the emergence of various techniques such as botnets [78]. In [77], a machine learning-based DDoS attack-detection mechanism is presented. This proposed solution enables the collection of IoT data, extracting the features and binary classification of IoT traffic to detect malicious traffic that initiates a DDoS attack. To build this mechanism, the authors used a variety of ML classifiers, namely random forests, K-nearest neighbors, support vector machines, decision trees, and neural networks.

Machine-learning algorithms have been also used for intrusion detection [79]. Zhao et al. [80] proposed a machine-learning-based intrusion-detection system that matches IoT characteristics requiring real-time monitoring. The authors based their solution on a dimension-reduction algorithm and a classifier. Principal Component Analysis (PCA) is used to decrease the size of the dataset of features to be analyzed. Furthermore, SoftMax regression and K-nearest are the two neighbor algorithms applied in the solution.

### 7.3. Blockchain in Smart IoT

Blockchain (BC) can be useful in many application fields, such as logistics and supply-chain management, Industry 4.0, the food industry, smart grid, and wireless network virtualization, to add more security features, to handle a large amount of data, and to support different components working together in a distributed decentralized network [81]. A decentralized BC platform can provide better protection in terms of security and privacy compared to the classical centralized architecture [82]. However, decentralized consensus algorithms suffer from high energy consumption and computing power, and cannot be implemented in IoT devices with limited resources and mobile edge servers. For instance, various frameworks based on BC have been proposed by exploiting built-in cryptography mechanisms and by combining a smart contract concept to enable the automated enforcement of some conditions in the real world [83,84]. In 5G applications communication systems and beyond, BC can enhance spectral efficiency and provide much better 5G traffic optimization while preserving privacy when different IoT devices share a link condition [85]. Despite all the advantages offered by BC technology and the related proposed frameworks based on it to improve security components, to the best of our knowledge there is no proposed framework that can provide a complete secured solution providing the confidentiality, integrity and availability (CIA) triad, preserving privacy, and offering multi-factor or remote authentication. Therefore, we believe that securing BC-based solutions for IoT is a big challenge for researchers in the future.

### 7.4. Securing 4G/5G and beyond Applications

Ferag and al. [86] presented a taxonomy of attacks against 4G/5G cellular networks based on four classes, including attacks against privacy, attacks against availability, attacks against integrity and attacks against authentication. Despite various countermeasures being provided to preserve privacy and authentication based on cryptography methods, human factors, and intrusion-detection systems to meet the security requirements for IoT in the 5G context, we believe that more research effort is necessary to achieve this goal. Some security issues related to the 5G network need to be resolved, such as the absence of a dataset for network intrusion detection in 5G scenarios. Furthermore, location and identity privacy are not preserved for 5G fog radio access network (F-RAN) and 5G cloud radio access network (C-RAN). Finally, recent research work regarding capacity extension of a massive MIMO channel [87] using new waveforms to enhance the performance of a 5G mobile system and to raise the number of connected IoT devices [88] needs to be enforced against privacy breaches and intrusion attacks in the C-RAN and F-RAN architecture.

## 8. Conclusions

In this paper, an IoT five-layer architecture is proposed based on potential security threats and countermeasures. Furthermore, the common attacks against IoT devices are exhibited, and the required countermeasures are reviewed. Indeed, IoT trends include securing the most relevant communication protocols, mitigating the security issues of the most important IoT platforms, and applications of the most important machine-learning trends to mitigate and predict security threats and risks. The main security features of IoT business platforms are addressed in terms of confidentiality, integrity, access control, authentication, secure communication, and encryption protocols. Finally, open research issues and future directions towards secure IoT devices and applications are discussed by providing standardized lightweight encryption algorithms, using machine-learning and blockchain, and enforcing security measures for 4G/5G mobile system applications and beyond.

## Figures and Tables

**Table 1:** Common attack against IoT devices according to the new architecture.

Layer	Common Attack	Description	Security Countermeasures
Data and Cloud services	Poisoning	input of incorrect training data/labels to decrease the accuracy of classification/clustering process	Data sanitization.
	Evasion	Generating an adversarial sample leading to evade system from detection spam and malware.	Retraining learning models by classifier designers with adversarial samples.
	Impersonate	Unauthorized access based on deep neural network DNN algorithm.	Defensive distillation on DNN.
	Inversion	Gathering information about ML models to compromise the data privacy.	Differential privacy (DP) technique and data encryption.
Application	Mirai malware	Gain access to IoT device by using a default Telnet or SSH account	Disabling/changing default account of Telnet and SSH account.
	IRCTelnet	Forcing Telnet port to infect LINUX operating system of IoT device.	Disabling Telnet port number.
	Injection	Untrusted data is sent to an interpreter as part of a command or query.	Input validation control.
Transport	TCP flooding	Sending many packets through TCP protocol to stop or to reduce his activities.	A classifier based on SVM to detect and prevent DDoS TCP flooding attack.
	UDP flooding	Sending a large number of packets through UDP protocol to stop or to reduce his activities.	A flow-based detection schema on router using a state machine and a hashing table.
	TCP SYN flooding	Tentative to open an externally connection without respecting to the TCP handshake procedure.	SYN-Cookies consist on coding client SYN message to change the state in the server side.
	TCP desynchronization	Tentative to break the packet sequence by injection a packet with a wrong sequence number.	Authentication for all packets in the TCP session.
Network/protocol	Man-in-the-middle	Violate the confidentiality and integrity in data transfer.	Intrusion-detection system (IDS) and virtual private network (VPN).
	DDoS	Making network resource unavailable for its intended use	Ingress/Egress filtering, D-WARD, Hop Count Filtering and SYN-Cookies.
	Replay	Manipulate the message stream and reorder the data packets.	Timeliness of Message.
Physical	Eavesdropping	Infer information sent by IoT devices via network	Faraday cage.
	Cyber-physical	Physically attacking a device	Use of fault-detection algorithm to identify the faulty nodes.
	RFID Tracking	to disable tags, modify their contents, or imitate them	Faraday cage.

**Table 2:** Most relevant IoT communication protocols.

Communication Protocol	*Standards*	*Encryption Protocol*	*Energy Consumption*	*Advantages*	*Disadvantages*
6LoWPAN	IEEE 802.15.4	AES	Low	Low processing	Lack of authentication
RPL	IETF RPL	AES	Low	Low processing	Vulnerability to many attacks
NFC	ISO/IEC 14443	RSA, DSA	Low	Simplicity of deployment	Limited Range
Bluetooth	IEEE 802.16	AES, ECDH	Medium/Very Low (BLE)	Low consumption	Privacy/Identity Tracking
Wi-Fi	IEEE 802.11i/e/g	AES	High	Mobility and efficiency	Limited reachability
Zigbee	IEEE 802.15.4	AES	Low	Low-cost, low-energy devices	one-time transmission of the unprotected key
WiMAX	IEEE 802.16	RSA	Medium	Supports authentication	Limited mobility
3G/4G/5G	UMTS/LTE	RSA, 3DES	Medium	Portability	Battery limitation

**Table 3:** SMQTT stack protocol.

OSI Layer	Protocol
Application	SMQTT
Session	SSL/TLS
Transport	TCP
Network	IPv4 and IPv6
Data-link	Ethernet/Wi-Fi

**Table 4:** Lightweight encryption algorithms for IoT.

	*Algorithm*	*Key Size*	*Execution Time*	*Application*
Symmetric	PRESENT	64 bits block with 80/128-bit length key	27.9	RFID
	CELFIA	128 bits block with 80/128/192 bits length key	-	Used by Sony for Digital Right Management
Asymmetric	RSA	1764 Bytes	19.33	Authentication
	Elliptic Curves	1272 Bytes	87.03	Pervasive Computing

**Table 5:** Security Features of Cloud-based IoT frameworks.

Cloud-Based IoT Framework	*Confidentiality*	*Integrity*	*Access Control*	*Authentication*	*Secure Communication*	*Encryption Protocol*
AWS IoT	SSL-protected, API endpoints	SSL-protected, API endpoints	Policy-based	X.509 certificates	SSL	TLS
Google IoT	ATLS	ATLS	Cloud IAM ACLs	ATLS RSA 2048	HTTPS, SSL	AES, 3DESTLS/S/MIME
Oracle IoT	SSL	PKI: Checksums	Roles-based	PKI: X.509 certificates, Kerberos	SSL	3DES, TSDP
CISCO IoT	IPsec	IPsec	Segment data based on destination	X.509 certificates	IPsec, TLS, MQTT over TLS	TLS, AES, RSA
Bosh IoT	WPA2	WPA2	No access control	SSID/Password	DTLS	LWM2M

**Table 6:** Machine-learning trends for IoT.

	*Algorithm*	*Complexity for Prediction*	*Advantages*	*Disadvantages*	*IoT Applications*
Classification	KNN	O(np)	Easy to update in online setting	Unscalable to large data sets	Smart Citizen, Smart Tourism
	Naive Bayes	O(p)	Fast and highly scalable	Strong feature independence assumptions	Smart Agriculture, Spam filtering, Text categorization
	SVM	O($n_{sv}p$)	Good for unbalanced data	The lack of transparency of results	Real-Time Prediction: Detection of Intrusion, attacks and malware.
Regression	Linear regression	O(p)	Processing under high rate	Very sensitive to outliers	Energy Applications, Market Prediction
	SVR	O($n_{sv}p$)	Useful and flexible technique	More complicated	Intelligent transportation systems, Smart Weather
Clustering	K-means	O(n2)	Very fast and highly scalable	Difficult to predict the number of clusters (K-Value)	Smart Cities, Smart Home, Smart Citizen, Intelligent Transport
	DBSCAN	O(n2)	fast and robust against outliers	Performance is sensitive to the distance metric	Smart Citizen, Smart Tourism
	Feed Forward Neural Network	O(n2)	Non-linearity and robustness	Longer time for training	Smart Health
